# Missed Infection-Control Nursing Care from the Early Pandemic to the Post-Pandemic Era: Policy and Management Implications for Safer Healthcare

**DOI:** 10.3390/healthcare14142077

**Published:** 2026-07-10

**Authors:** Eftychia Evangelidou, Evridiki Papastavrou, Georgios Efstathiou, Chryssoula Lemonidou

**Affiliations:** 1 Deputy Director of Nursing Directorate, General Hospital of Nea Ionia “Konstantopoulio–Patision”, 14233 Athens, Greece; 2 Department of Nursing, Cyprus University of Technology, Limassol 3036, Cyprus; e.papastavrou@cut.ac.cy; 3 Educational Sector, Nursing Services, Ministry of Health, Limassol 2049, Cyprus; 4 Department of Nursing, National and Kapodistrian University of Athens, 11527 Athens, Greece

**Keywords:** missed nursing care, infection prevention and control, patient safety, healthcare policy, nurse staffing, healthcare-associated infections, post-pandemic healthcare

## Abstract

Background: Missed nursing care related to infection prevention and control compromises patient safety and reflects clinical practice gaps and organizational constraints. The COVID-19 pandemic intensified awareness of infection-control practices; however, whether this translated into sustained reductions remains unclear. Aim: To compare missed infection-control nursing care between the pre-/early-pandemic period (2019–2020) and the post-pandemic period (2026) and identify persistent omissions with implications for healthcare policy and management. Methods: A descriptive study was conducted among 1570 nurses, including 774 participants in Group A (2019–2020) and 796 in Group B (2026). Data were collected online using the Missed Infection Control Nursing Care Questionnaire, tested for reliability and validity in Greek. Data were analyzed using SPSS 25.0, with statistical significance set at α = 0.05. Results: Item-level analysis showed lower mean omission scores in 34/37 infection-control nursing care practices, with 26 statistically significant reductions. The largest decreases were observed for glove use during antibiotic preparation/administration (1.493 to 1.070), hand hygiene before medication administration (1.340 to 0.962), multidrug-resistant organism (MDRO) admission screening (1.849 to 1.481), and intravenous access hub disinfection (1.978 to 1.668). In 2026, key residual omissions involved urinary catheter care (31.2%), hub disinfection (33.2%), oral hygiene (30.9%), and environmental hygiene before meals (29.1%). Conclusions: Missed infection-control nursing care declined in the post-pandemic period, but system-dependent omissions persisted, highlighting the need for staffing adequacy, balanced workload allocation, environmental support, and routine integration of infection-prevention practices.

## 1. Introduction

Healthcare-associated infections (HAIs) remain one of the most persistent and preventable threats to patient safety. Beyond their immediate clinical consequences, HAIs increase length of stay, healthcare costs, antimicrobial use and the risk of antimicrobial resistance, placing pressure on both patients and health systems. Infection prevention and control (IPC) is therefore not a narrow technical field, but a core component of quality, safety and health system resilience [1]. The World Health Organization has explicitly positioned IPC at the center of patient safety, health worker protection, antimicrobial resistance control, outbreak preparedness and health system strengthening [2,3]. Similarly, the European Centre for Disease Prevention and Control highlights that a substantial proportion of HAIs are preventable through effective IPC measures, reinforcing the need for reliable implementation in everyday clinical practice [4].

Nurses have a central role in IPC because many prevention practices are embedded in routine nursing care. Hand hygiene, use of personal protective equipment, catheter care, oral hygiene, environmental hygiene, surveillance of infection signs, patient education and adherence to isolation precautions are not isolated procedures; they are repeated bedside actions that require time, attention, staffing, supplies and coordination [5]. When these actions are delayed, incomplete or omitted, missed nursing care becomes directly connected with infection risk. The concept of missed, rationed, unfinished or omitted nursing care has gained increasing attention in recent years because it captures the gap between care that is required and care that is actually delivered [6,7]. This gap is particularly important for IPC, where small omissions can accumulate and undermine patient safety.

The COVID-19 pandemic brought IPC into the foreground of healthcare delivery. During the pandemic, healthcare systems intensified attention to hand hygiene, personal protective equipment, isolation, environmental cleaning and staff education. In many settings, IPC was no longer perceived as the responsibility of infection-control teams alone, but as an everyday concern for all healthcare workers. Although IPC awareness increased substantially during the pandemic, it remains unclear whether these improvements have been sustained over time or whether persistent organizational pressures continue to contribute to missed infection-control nursing care [8,9,10]. Recent evidence suggests that missed and unfinished nursing care persisted during the pandemic and was strongly shaped by labor resources, workload, patient acuity and organizational constraints [8,9,10].

This issue is not only clinical, but also managerial and political. Missed IPC nursing care should not be interpreted simply as a matter of individual compliance or professional responsibility. Although education, knowledge and adherence to guidelines are important, nurses deliver IPC care within systems that may or may not enable safe practice [11]. Staffing levels, skill mix, workload distribution, support staff availability, patient flow, isolation capacity, cleaning services, supplies and leadership support all influence whether IPC tasks can be completed consistently. Evidence from the wider missed-care literature shows that nurse staffing and work environment are closely related to missed care and patient outcomes [8,12]. For this reason, missed IPC nursing care is a useful indicator not only of bedside practice but also of the organizational conditions under which nurses work.

Recent IPC-specific studies have begun to address this gap. Moreal et al. (2024) compared missed IPC activities before and after the pandemic and reported evidence of post-pandemic change, suggesting that lessons from COVID-19 may have influenced IPC practice [13]. Kirwan and Egan (2025) also examined missed IPC practices in acute hospitals during the COVID-19 pandemic and highlighted the need to understand both nurses’ perceptions and the organizational capacity of hospitals to support IPC work [14].

A single global missed-care score may hide clinically meaningful differences between practices. For example, hand hygiene before medication administration, urinary catheter care, oral hygiene, intravenous access hub disinfection, environmental cleaning and MDRO admission screening may not change in the same way or for the same reasons. Some practices are highly visible and strongly reinforced through pandemic-era messaging, whereas others are more time-dependent, less visible and more sensitive to workload and staffing. Identifying these differences is essential for healthcare managers because interventions must target the specific practices and system barriers that remain problematic [13,14].

The aim of the present study, therefore, was to compare missed infection-control nursing care between the 2019–2020 and 2026 periods using an item-level analysis. The study focuses on two related questions: first, whether reported omissions in specific IPC nursing care practices differed between the two periods, and second, which organizational barriers remained important in the post-pandemic period. By linking missed IPC care with healthcare policy and management, the study aimed to contribute evidence that can inform staffing policies, workload allocation, environmental support, IPC monitoring and ward-level quality improvement strategies.

The baseline findings from the initial 2019–2020 phase have been reported previously in a national peer-reviewed publication using descriptive analyses. The present study extends that work by incorporating an independent post-pandemic cohort, enabling direct comparison between two study periods, multivariable adjusted analyses, and a policy-oriented interpretation of persistent infection-control nursing care omissions.

## 2. Methods

### 2.1. Study Design

A repeated comparative cross-sectional study was conducted using two independent samples collected during two separate data-collection phases. The first phase corresponded to the 2019–2020 period (Group A), whereas the second phase was conducted during 2026 (Group B). As different participants were recruited in each phase, the study does not represent a longitudinal follow-up of the same individuals.

### 2.2. Participants and Sampling

The study population consisted of nurses working in different clinical departments of public and private hospitals in Greece and Cyprus. Participants were recruited through convenience sampling. The questionnaire was disseminated electronically through professional nursing networks and institutional communication channels in Greece and Cyprus. Because participation was voluntary and the survey was distributed through multiple online dissemination pathways, the total number of nurses who received the invitation could not be determined. Consequently, a response rate could not be calculated. Eligible participants were registered nurses who were employed in hospital settings, provided direct nursing care to hospitalized patients, and agreed to participate voluntarily in the study.

To ensure that the sample size was adequate for the planned analyses, an a priori power analysis was performed using G*Power software, version 3.1.9.2 (Heinrich Heine University Düsseldorf, Germany). The calculation was based on a significance level of α = 0.05 and statistical power of 0.95. The minimum required sample size was estimated at 741 participants, according to the assumptions used in the power analysis approach described by Faul et al. [15]. The final sample exceeded this requirement.

### 2.3. Instrument

Data were collected using the 62-item Missed Infection Control Nursing Care Questionnaire, originally developed by Blackman et al. (2017) [16]. Permission to use the instrument was obtained from the developer prior to data collection.

The questionnaire includes three sections. The first section collects demographic and professional information. The second section assesses infection-control nursing care practices that may be missed during routine clinical care. This section consists of 37 items rated on a six-point Likert-type scale, ranging from “never missed” to “always missed”, also with the option of “unknown/not applicable”. The third section explores possible reasons for missed infection-control nursing care and includes 24 items rated on a five-point Likert-type scale, ranging from “not an important reason” to “important reason”, also with the option of “unknown/not applicable”. Together, these sections provide information not only on which infection-control practices are omitted but also on the organizational and work-related factors that may contribute to these omissions.

For use in the Greek-speaking context, the questionnaire was translated and culturally adapted through a forward–backward translation procedure. Content validity was then assessed by an expert panel consisting of eight academics in nursing, four from Greece and four from Cyprus. Following this review, the instrument was pilot tested with 20 nurses who had characteristics similar to those of the target population. The pilot phase aimed to evaluate the clarity, comprehensibility, possible ambiguities, and approximate time needed for completion. No major comprehension difficulties were identified, and no content modifications were required after the pilot testing process, in line with recommended procedures for instrument evaluation [17].

The content validity index confirmed satisfactory. For Part B, the item-level content validity index (I-CVI) ranged from 0.875 to 1.00, while the scale-level content validity index based on universal agreement (S-CVI/UA) was 0.89. For Part C, the I-CVI ranged from 0.75 to 1.00, and the S-CVI/UA was 0.84.

### 2.4. Data Collection

Data were collected electronically through an online survey platform. The first phase of data collection took place from 2019 to 2020, a period that coincided with the emergence of the first international reports and early public health responses related to SARS-CoV-2. The second phase was conducted during 2026, allowing comparison with a later post-pandemic period. Participation was voluntary, and the questionnaire was completed anonymously.

### 2.5. Statistical Analysis

Data were analyzed using IBM SPSS Statistics version 25.0 (IBM Corp., Armonk, New York, NY, USA). Statistical significance was set at α = 0.05, and all tests were two-tailed. The analysis compared two study periods: 2019–2020 and 2026. Because the analyses were based on two independent cross-sectional samples, comparisons between study periods were interpreted as associations between cohorts rather than as evidence of temporal or causal effects.

Descriptive statistics were used to summarize demographic and professional characteristics. Continuous variables were represented as mean ± standard deviation and median with interquartile range, while categorical variables were represented as frequencies and percentages. Differences in baseline characteristics between the two periods were assessed using appropriate comparative tests: the independent samples *t*-test or Mann–Whitney U test for continuous variables, and the chi-square test or Fisher’s exact test for categorical variables.

Missed infection-control nursing care was analyzed at the item level. Each care item was scored from 0 to 4, with higher values indicating more frequent omission. Responses recorded as “unknown” or “not applicable” were treated as missing for the corresponding item and excluded from item-specific analyses. The frequency of “unknown/not applicable” responses was examined for key infection-control nursing care items to improve transparency. These responses were excluded from comparative analyses because they reflected non-applicable clinical situations rather than omitted nursing care. For each item, mean omission scores were calculated for both periods and compared between 2019–2020 and 2026. In addition, item responses were dichotomized to identify clinically relevant omissions, defined as care reported as “frequently” or “always” missed. The proportion of frequent/always missed care was calculated for each item and compared between periods.

To examine whether differences between periods remained after adjustment for participant and work-related characteristics, multivariable logistic regression models were fitted for selected high-priority infection-control nursing care items. For each model, the dependent variable was frequent/always missed care for the specific item. The main independent variable was study period, with 2019–2020 used as the reference group. Models were adjusted for age, sex, country of work, workplace sector, postgraduate education, specialized infection-control duties, previous infection-prevention and control training, current workplace experience greater than 10 years, overtime during the previous three months, and perceived impact of insufficient staffing. These covariates were selected a priori based on their clinical relevance, previous literature, and their potential role as confounding variables. Variable selection was not based on statistical significance in bivariable analyses.

Adjusted odds ratios with 95% confidence intervals were reported. An adjusted odds ratio below 1 indicated lower odds of frequent/always missed care in 2026 compared with 2019–2020. Prior to model fitting, multicollinearity between the independent variables was assessed using variance inflation factors (VIFs). No evidence of problematic multicollinearity was identified, with all calculated VIF values below 2.0.

### 2.6. Ethical Considerations

The study protocol was submitted for ethical review and approval to the Ethical Committee of National and Kapodistrian University of Athens, Department of Nursing (Ref. No. 2833/16-07-2019), and to the Cyprus National Bioethics Committee (Ref. No. EEBK EP 2019.01.21).

Before completing the questionnaire, participants received detailed information about the study through an introductory information sheet that accompanied the online survey. This information explained the purpose of the study, the voluntary nature of participation, and the anonymous handling of responses. Completion and submission of the questionnaire were considered to indicate informed consent to participate.

All data were stored securely and were accessible only to the research team. Throughout the study process, confidentiality and anonymity were maintained, and no information that could identify individual participants was collected or reported.

## 3. Results

### 3.1. Sample Characteristics

A total of 1570 valid responses, comprising 774 nurses in Group A and 796 nurses in Group B, were included in the analysis. The two groups differed in several baseline characteristics. Participants in Group B were older than those in Group A (47.0 ± 9.3 vs. 40.3 ± 8.7 years, *p* < 0.001), and a higher proportion were female (87.7% vs. 80.5%, *p* < 0.001). No statistically significant differences were observed for postgraduate education, specialized infection-control duties, previous infection-prevention and control training, current workplace experience greater than 10 years, or overtime (Table 1).

### 3.2. Item-Level Changes in Missed Infection-Control Nursing Care

Missed infection-control nursing care was examined at the individual item level rather than as a total composite score. Each item was scored from 0 to 4, where higher values indicate more frequent omission. Responses of “unknown/not applicable” were treated as missing for the specific item.

Thirty-four of the 37 infection-control nursing care items showed lower mean omission scores in Group B compared with Group A. Of these, 26 items showed statistically significant reductions. No item showed a statistically significant increase in missed care.

The largest reductions were observed for wearing gloves during antibiotic preparation/administration, hand hygiene before medication administration, screening new admissions for MDRO infection or colonization, use of eye/mask/face-shield protection for airborne or droplet precautions, and disinfection of intravenous access hubs (Table 2).

### 3.3. Residual Missed Infection-Control Nursing Care in Group B

Despite the overall pattern of improvement across most individual items, several practices continued to show relatively high omission scores in Group B. The highest residual omissions were observed in practices that are strongly dependent on workload, workflow organization, environmental support, and systematic surveillance.

The highest mean omission score in Group B was observed for urinary catheter care every 8 h (mean = 1.745; 31.2% frequent/always omitted), followed by disinfection of intravenous access hubs (mean = 1.668; 33.2% frequent/always omitted), cleaning the bedside table before meal delivery (mean = 1.606; 29.1% frequent/always omitted), daily oral hygiene (mean = 1.603; 30.9% frequent/always omitted), and screening new admissions for MDRO infection or colonization (mean = 1.481; 27.8% frequent/always omitted) (Table 3). For the key infection-control nursing care items discussed above, the proportion of responses recorded as “unknown/not applicable” ranged from 8.8% for intravenous access hub disinfection to 22.6% for MDRO admission screening. Higher proportions of non-applicable responses were also observed for urinary catheter care (21.6%) and oral hygiene (15.5%), reflecting that these practices were not relevant to all participants’ clinical settings. Consequently, these responses were excluded from item-level comparative analyses.

### 3.4. Reported Reasons for Missed Infection-Control Nursing Care

In Group B, the highest-rated reasons were predominantly organizational and staffing-related. The most prominent were inadequate nurse/support staff ratio for patient care assignment (mean = 2.670; 79.7% reported as an important reason), insufficient nursing staff (mean = 2.669; 81.0% important reason), patients sharing the same bathroom (mean = 2.643; 76.6% important reason), unbalanced patient assignment (mean = 2.619; 75.1% important reason), and insufficient cleaning/support staff (mean = 2.575; 71.9% important reason) (Table 4).

### 3.5. Changes in Reported Reasons Between Periods

The largest increases between Group A and Group B were observed for insufficient clerical support (mean difference = +0.198, *p* = 0.002), insufficient physicians (+0.179, *p* = 0.002), unexpected increase in patient volume or acuity (+0.136, *p* = 0.003), and insufficient storage for personal items (+0.134, *p* = 0.013).

In contrast, several management- or monitoring-related barriers decreased. The largest decreases were observed for lack of hospital management support for resources (−0.149, *p* = 0.002), lack of nursing monitoring of IPC actions (−0.113, *p* = 0.024), and lack of management support for Infection Control Committees (−0.106, *p* = 0.053) (Table 5).

### 3.6. Item-Level Regression Analysis

To examine whether the observed differences between the two study periods persisted after accounting for demographic, professional, and organizational characteristics, multivariable logistic regression models were fitted for selected clinically important infection-control nursing care items.

Because missed infection-control nursing care was analyzed at the individual item level, each regression model was conducted separately for one specific infection-control nursing care practice. For each model, the dependent variable was whether the specific care practice was reported as frequently or always omitted. Responses of “never”, “rarely”, or “occasionally” omitted were grouped as the reference category.

The main independent variable was study period, comparing Group B with Group A, with Group A used as the reference category. Models were adjusted for age, sex, country of work, workplace sector, postgraduate education, specialized infection-control duties, previous infection-prevention and control training, current workplace experience greater than 10 years, overtime during the previous three months, and perceived impact of insufficient staffing.

Thus, the adjusted odds ratio represents the odds of reporting a specific infection-control nursing care practice as frequently or always omitted in Group B compared with Group A after controlling for demographic, professional, and work-related characteristics. An adjusted odds ratio below 1 indicates lower odds of frequent/always omission in Group B.

In the adjusted models, Group B was associated with significantly lower odds of frequent/always omission for several infection-control nursing care practices. These included screening new admissions for multidrug-resistant organism infection or colonization (aOR = 0.56, 95% CI 0.41–0.76, *p* < 0.001), wearing gloves during antibiotic preparation/administration (aOR = 0.53, 95% CI 0.39–0.73, *p* < 0.001), eye/mask/face-shield protection for airborne or droplet precautions (aOR = 0.66, 95% CI 0.47–0.94, *p* = 0.023), cleaning/support staff wearing appropriate personal protective equipment (aOR = 0.58, 95% CI 0.39–0.87, *p* = 0.008), hand hygiene before medication administration (aOR = 0.70, 95% CI 0.50–0.98, *p* = 0.036), and urinary catheter care every 8 h (aOR = 0.72, 95% CI 0.53–0.98, *p* = 0.037).

For other clinically relevant residual omissions, including disinfection of intravenous access hubs, cleaning the bedside table before meal delivery, oral hygiene at least once daily, and patient hand hygiene after bedpan/urinal use, the adjusted associations were not statistically significant, although crude omission percentages were lower in Group B (Table 6).

The regression findings indicate that after adjustment for participant and work-related characteristics, Group B had lower odds of frequent/always omission in several visible and protocol-driven infection-control practices, particularly MDRO admission screening, glove use during antibiotic preparation/administration, PPE use for airborne or droplet precautions, hand hygiene before medication administration, cleaning/support staff PPE use, and urinary catheter care. However, some persistent omissions, including intravenous access hub disinfection, oral hygiene, bedside environmental cleaning, and patient hand hygiene after bedpan/urinal use, did not remain statistically significant after adjustment.

## 4. Discussion

The present study provides a comparative, item-level view of missed infection-control nursing care between the 2019–2020 and 2026 periods. Because the study compared two independent cross-sectional samples, the observed differences between the two study periods should be interpreted as associations rather than direct effects of the COVID-19 pandemic. In addition to pandemic-related changes, broader developments in healthcare delivery, infection prevention and control practices, organizational priorities, workforce composition, and other unmeasured contextual factors may have also contributed to the observed differences. Rather than treating missed infection-control care as a single global score, the analysis highlights which specific practices improved and which remained vulnerable. This is important because infection prevention and control (IPC) is not one behavior; it is a bundle of repeated bedside, environmental, surveillance and organizational actions that must be completed reliably in everyday clinical work. These findings should be interpreted within the context of a repeated comparative cross-sectional design involving two independent cohorts. Consequently, differences observed between the study periods may reflect not only changes over time but also differences in participant characteristics and healthcare settings despite statistical adjustment for measured covariates.

In addition, the two cohorts differed in several baseline characteristics, including age, sex, and workplace sector. Although these variables were included in the adjusted regression models, residual differences between the cohorts may still have contributed to the observed variation in missed infection-control nursing care. Consequently, the findings should be interpreted with appropriate caution when comparing the two study periods.

The findings suggest a broad improvement in reported missed IPC nursing care in Group B. Most individual care items showed lower omission scores compared with Group A, with the largest reductions observed in visible and protocol-driven practices such as glove use during antibiotic preparation/administration, hand hygiene before medication administration, MDRO admission screening, use of eye/mask/face-shield protection for airborne or droplet precautions, and disinfection of intravenous access hubs. This pattern is consistent with Moreal et al., who found that missed IPC activities were less likely in the post-pandemic period and argued that pandemic-related system efforts may have helped routinize IPC practices among nurses [13].

At the same time, the findings should not be interpreted as complete and sustained improvement. Several clinically important IPC practices remained frequently omitted in Group B, including urinary catheter care, intravenous access hub disinfection, oral hygiene, environmental cleaning before meal delivery, and MDRO screening at admission. This finding has important implications for healthcare policy and management. These activities are not simply matters of individual compliance; they require time, staffing, clear workflow, supplies, documentation routines, environmental support and coordination across professional groups. The persistence of these omissions echoes the wider missed-care literature, where basic care, surveillance activities and time-dependent tasks are often the first to be compromised under workload pressure [5,6,18].

The persistence of omissions in urinary catheter care, oral hygiene, and intravenous access hub disinfection is not unexpected, as these activities are repetitive, time-dependent, and integrated into routine bedside care rather than highly visible IPC behaviors. Consequently, they are more vulnerable to competing clinical priorities, workflow interruptions, and staffing constraints. Previous studies have similarly shown that workload, inadequate staffing, and unfavorable work environments disproportionately affect fundamental nursing activities requiring continuous surveillance and repeated bedside interventions [11,18,19,20].

From a healthcare management perspective, reducing these persistent omissions is likely to require system-level interventions beyond staff education alone. Workflow redesign, structured audit and feedback, standardized care pathways, and regular monitoring of high-risk IPC practices may improve the consistent delivery of these interventions, particularly in resource-constrained environments [18,20,21].

The post-pandemic improvement observed in this study also fits with the idea that COVID-19 made IPC more visible in clinical settings. During the pandemic, healthcare organizations repeatedly reinforced hand hygiene, PPE use, isolation procedures, environmental cleaning and staff education. These practices became part of the everyday language of healthcare delivery. However, our findings show that visibility alone is not enough. Highly visible behaviors may improve, while less visible but equally important nursing activities—such as oral hygiene, catheter care or systematic environmental preparation—may remain vulnerable when wards are understaffed or under-resourced.

This interpretation is supported by evidence from studies of missed and unfinished nursing care during COVID-19. A comparative cross-sectional study in critical care reported that missed nursing care remained a patient safety concern before and during the pandemic, with basic care frequently affected [19]. Another study similarly showed that unfinished nursing care during the pandemic was strongly linked to labor resources, workload, patient acuity and nurses’ psychological burden [22]. Labrague and Kostovich also concluded that despite the unique conditions of the pandemic, inadequate nursing workforce remained the most consistent reason for missed care [18].

A particularly important finding of the present study is that the main reasons for missed IPC care in Group B were organizational. Insufficient nursing staff, inadequate nurse/support staff ratios, unbalanced patient assignments, shared bathrooms and insufficient cleaning/support staff were among the highest-rated barriers. These findings suggest that organizational factors may contribute to missed infection-control nursing care, and therefore, have important implications for healthcare policy and management. The results suggest that missed IPC nursing care should not be framed solely as a matter of individual knowledge, motivation, or compliance. Rather, the findings are consistent with the possibility that organizational conditions, including staffing, work environment, and support services, may influence the delivery of infection-prevention practices. However, these factors were not directly measured in the present study and should therefore be interpreted cautiously.

This is consistent with the broader evidence linking staffing, work environment and missed care. Dall’Ora et al., in a systematic review of longitudinal studies, found that higher registered nurse staffing was generally associated with better patient outcomes, particularly mortality, although evidence varied by outcome [21]. Nantsupawat et al. reported associations between poor nurse staffing, missed care, lower quality of care and adverse events [20]. Lake et al. also showed that improved work environments and staffing were associated with less missed nursing care [11]. Together, these findings support the use of missed care as a sensitive indicator of whether staffing and work organization are sufficient to deliver safe care.

From an IPC policy perspective, this is especially relevant. Healthcare-associated infections remain a major source of avoidable harm, cost and antimicrobial resistance. The World Health Organization has positioned IPC as central to patient safety, health worker protection, outbreak preparedness and antimicrobial resistance control [2,3]. The WHO Global Strategy on IPC calls for action at national, subnational and facility levels, while the WHO minimum requirements for IPC programs emphasize that facilities need functioning IPC programs, trained teams, surveillance, education, monitoring, multimodal strategies and adequate infrastructure [2,3].

The European picture reinforces this urgency. The ECDC states that up to 50% of healthcare-associated infections are estimated to be preventable through effective IPC measures and highlights the contribution of HAIs to infections caused by antibiotic-resistant bacteria [4]. Therefore, when nursing activities such as catheter care, hub disinfection, oral hygiene, MDRO screening or environmental hygiene are missed, the issue is not minor or administrative. It directly concerns preventable harm, antimicrobial resistance and the sustainability of healthcare systems.

The present study therefore contributes a practical message: post-pandemic IPC awareness may have improved some practices, but policies must now move from awareness to reliability. In other words, the question is no longer only whether nurses know what should be done. The findings suggest that sustained delivery of infection-prevention practices may depend on whether healthcare systems provide nurses with adequate staffing, time, support, equipment, and workflow conditions. This distinction matters. Education and guidelines are necessary, but they will not be sufficient if nurses are working in environments where workload, patient acuity, admissions, discharges and inadequate support services make complete IPC care unrealistic.

The item-level regression findings strengthen this interpretation. After adjustments for demographic, professional and organizational characteristics, Group B was associated with lower odds of frequent/always omission for several IPC practices, including MDRO admission screening, glove use during antibiotic preparation/administration, PPE use for airborne or droplet precautions, cleaning/support staff PPE use, hand hygiene before medication administration and urinary catheter care. This suggests that the observed reductions were not explained only by differences in participant characteristics between the two periods. However, other important practices, including intravenous access hub disinfection, oral hygiene and bedside environmental cleaning, did not remain statistically significant in adjusted models. This may indicate that these practices are particularly sensitive to unmeasured ward-level conditions such as patient acuity, staffing per shift, isolation capacity, cleaning availability, interruptions, admissions/discharges and local IPC culture.

These findings align with recent IPC-specific evidence. Kirwan and Egan (2025) examined missed IPC practices in acute hospitals in Ireland during the COVID-19 pandemic and underlined the importance of understanding both nurses’ perceptions of missed IPC practices and hospitals’ capacity to support IPC work [14]. Moreal et al. similarly emphasized that predictors of missed IPC care changed after the pandemic and that new interventions may need to target unit-level patterns [13]. Our findings add to this literature by showing that even when many individual IPC practices improve, the remaining gaps cluster around activities that require organizational reliability, not only individual vigilance.

For healthcare managers, the implications are direct. First, staffing policies should treat IPC nursing care as a core workload component, not as an additional task that can be absorbed into already full shifts. Second, patient assignment should consider infection risk, isolation needs, device care, oral care requirements and MDRO screening workload, not only patient numbers. Third, support services—especially cleaning staff, clerical support and supply availability—should be included in IPC planning. Fourth, IPC indicators should be embedded into ward-level dashboards, with feedback on specific missed practices rather than only broad compliance indicators. Finally, nurse managers and IPC teams should jointly review which practices are most often missed and redesign workflows accordingly.

This has broader policy relevance. Global IPC strategies increasingly emphasize implementation, monitoring and accountability rather than guidance alone [2,3]. The present findings are consistent with this broader direction. The present findings suggest that future policy initiatives may benefit from considering safe staffing frameworks, minimum IPC staffing and infrastructure standards, routine audit of high-risk nursing IPC practices, escalation pathways when IPC care is missed, and stronger integration of IPC into quality-management systems. Without these structural supports, post-pandemic gains may fade as health systems return to chronic workload pressure and resource constraints.

The study suggests that missed IPC nursing care was lower in Group B compared with Group A across many individual practices, but the remaining omissions are clinically meaningful and policy-relevant. The main message is not simply that nurses omitted less care after the pandemic. The stronger message is that sustainable IPC depends on whether healthcare systems can convert pandemic-era awareness into reliable, resourced, ward-level practice.

### Study Limitations

Several limitations of the study should be recognized. First, the study employed a repeated comparative cross-sectional design using two independent samples rather than a longitudinal follow-up of the same participants. Consequently, the observed differences between the 2019–2020 and 2026 periods should be interpreted as associations between two independent cohorts and cannot be attributed exclusively to temporal changes or the long-term effects of the COVID-19 pandemic. Although the multivariable models were adjusted for several participant characteristics, unmeasured differences between the cohorts may have contributed to the observed findings.

Second, the data relied on self-reported information. Although this approach is suitable for capturing nurses’ perceptions of omitted care, it is susceptible to biases such as recall bias, social desirability bias, and variations in professional awareness. For instance, nurses with higher IPC expertise or specialized roles might be more attuned to omissions, and thus, more likely to report them, even if their actual care practices do not differ significantly. Furthermore, self-reported assessments of infection prevention and control practices may be influenced by social desirability bias, whereby participants tend to overestimate their adherence to recommended practices. Consequently, the reported omission rates may not fully reflect actual clinical practice.

Third, there were baseline differences between the two comparison groups, including factors such as age, sex, country of employment, and workplace sector. While the adjusted models accounted for selected demographic, professional, and organizational variables, the possibility of residual confounding remains. In addition, participants were recruited using convenience sampling, which may have introduced selection bias and limits the representativeness and generalizability of the findings. Furthermore, because the survey was disseminated through multiple electronic distribution channels without a predefined sampling frame, the total number of nurses who received the invitation could not be determined; therefore, a response rate could not be calculated. In addition, hospital-level characteristics, such as hospital size, teaching status, and geographical region, were not collected, and therefore, could not be examined as potential sources of variation or selection bias.

Additionally, the analysis was conducted at the item level because no validated composite missed-care score was predetermined as the primary outcome. Although this approach allows for maintaining the clinical specificity of each IPC practice, it prevents summarizing missed IPC care into a single overarching measure.

Another limitation lies in the missing or not-applicable responses for certain items, which reduced the sample size available for specific analyses. This could have impacted both the precision and statistical power of some item-level comparisons and regression models.

Lastly, the study did not incorporate objective patient-level or ward-level outcomes, such as rates of healthcare-associated infections (HAIs), catheter-associated urinary tract infections, bloodstream infections, multidrug-resistant organism (MDRO) transmission, antimicrobial resistance metrics, nurse-to-patient ratios during shifts, bed occupancy rates, patient acuity levels, or the availability of cleaning staff. Therefore, the findings reflect nurses’ perceptions of missed infection-control nursing care rather than objectively observed clinical practice and should be interpreted alongside objective infection surveillance measures whenever such data are available. Future research should aim to establish links between missed IPC nursing care and these objective indicators. Future research should also consider combining questionnaire-based assessments with direct observational audits of infection-prevention practices, thereby enabling triangulation between perceived and observed clinical performance.

## 5. Conclusions

This study found that many missed infection-control nursing care practices were reported less frequently in 2026 than in 2019–2020. The strongest improvements were seen in visible and protocol-driven IPC practices, including PPE-related behaviors, hand hygiene before medication administration, antibiotic-related glove use and MDRO admission screening. However, important omissions persisted in urinary catheter care, intravenous access hub disinfection, oral hygiene, environmental hygiene before meal delivery and MDRO screening. These practices appear to be closely associated with staffing, workload, support services, environmental resources, and ward-level organization.

The findings of the present study suggest that missed infection-control nursing care may be more effectively addressed through system-level quality and safety approaches rather than relying solely on education or individual compliance. These findings support consideration of policies that prioritize safe staffing, workload-based patient allocation, sufficient cleaning and support staff, reliable access to supplies, appropriate environmental infrastructure, and routine monitoring of high-risk infection-prevention and control nursing activities.

Future studies should use longitudinal and multicenter designs; combine self-reported missed care with objective infection surveillance data; and examine ward-level predictors such as nurse-to-patient ratios, patient acuity, admissions and discharges, isolation capacity, cleaning resources and IPC audit feedback. Intervention studies are also needed to test whether staffing redesign, IPC checklists, electronic reminders, structured MDRO screening pathways and ward-level feedback can reduce persistent missed IPC nursing care and improve patient safety outcomes.

## Figures and Tables

**Table 1:** Baseline characteristics.

Characteristic	Group A	Group B	*p*-Value
Age, years (mean ± SD)	40.3 ± 8.7; 40 [34–47]	47.0 ± 9.3; 47 [42–55]	<0.001
N (%)			
Female sex	607 (80.5%)	691 (87.7%)	<0.001
Public workplace	687 (89.6%)	769 (97.0%)	<0.001
Postgraduate education	352 (45.5%)	391 (49.1%)	0.163
Specialized IPC duties	67 (9.0%)	62 (7.9%)	0.488
Previous IPC training	656 (99.5%)	693 (99.4%)	1.000
Current workplace experience > 10 years	420 (57.0%)	474 (61.2%)	0.110
Overtime last 3 months: none	253 (34.2%)	271 (34.8%)	0.093
Overtime last 3 months: 1–4 h	153 (20.7%)	192 (24.7%)	0.093
Overtime last 3 months: 5–10 h	126 (17.0%)	135 (17.4%)	0.093
Overtime last 3 months: >11 h	208 (28.1%)	180 (23.1%)	0.093

**Table 2:** Infection-control nursing care items with the largest reductions between Group A and Group B.

Item	Group A Mean	Group B Mean	Mean Difference	Frequent/Always Omitted Group A (%)	Frequent/Always Omitted Group B (%)	*p*-Value
Gloves during antibiotic preparation/administration	1.493	1.070	−0.422	28.5%	15.6%	<0.001
Hand hygiene before medication administration	1.340	0.962	−0.378	22.0%	12.6%	<0.001
Screening new admissions for MDRO infection/colonization	1.849	1.481	−0.368	41.3%	27.8%	<0.001
Eye/mask/face shield for airborne or droplet precautions	1.227	0.898	−0.329	20.8%	13.6%	<0.001
Disinfection of intravenous access hubs	1.978	1.668	−0.309	42.2%	33.2%	<0.001
Cleaning/support staff wearing appropriate PPE	1.083	0.800	−0.284	17.4%	9.7%	<0.001
Cleaning bedside table before meal tray	1.882	1.606	−0.277	39.0%	29.1%	<0.001
Cleaning/support staff compliance with precaution signage	1.051	0.786	−0.264	15.9%	8.8%	<0.001
Appropriate signage for standard/transmission-based precautions	0.867	0.628	−0.239	14.3%	7.6%	<0.001
Hand hygiene before patient contact	1.289	1.063	−0.225	21.7%	15.4%	<0.001

Mean difference = Group B minus Group A. Negative values indicate lower reported omission frequency in Group B.

**Table 3:** Highest residual missed infection-control nursing care items in Group B.

Item	Group A Mean	Group B Mean	Mean Difference	Frequent/Always Omitted Group B (%)	*p*-Value
Urinary catheter care every 8 h	1.965	1.745	−0.220	31.2%	0.004
Disinfection of intravenous access hubs	1.978	1.668	−0.309	33.2%	<0.001
Cleaning bedside table before meal tray	1.882	1.606	−0.277	29.1%	<0.001
Oral hygiene at least once daily	1.685	1.603	−0.081	30.9%	0.307
Screening new admissions for MDRO infection/colonization	1.849	1.481	−0.368	27.8%	<0.001
Patient hand hygiene after bedpan/urinal use	1.403	1.179	−0.224	18.3%	0.001
Preoperative bathing	1.099	1.103	0.004	18.2%	0.956
Gloves during antibiotic preparation/administration	1.493	1.070	−0.422	15.6%	<0.001
Hand hygiene before patient contact	1.289	1.063	−0.225	15.4%	<0.001
Using nursing observations to suggest further tests	1.092	0.973	−0.119	11.7%	0.059

**Table 4:** Comparison of the main reported reasons for missed infection-control nursing care between Group A and Group B.

Reason	Group A Mean	Group B Mean	Mean Difference	Important Reason Group B (%)	*p*-Value
Inadequate nurse/support staff ratio for patient care assignment	2.647	2.670	0.023	79.7%	0.552
Insufficient nursing staff	2.602	2.669	0.067	81.0%	0.101
Patients sharing the same bathroom	2.635	2.643	0.008	76.6%	0.842
Unbalanced patient assignment	2.609	2.619	0.011	75.1%	0.790
Insufficient cleaning/support staff	2.518	2.575	0.057	71.9%	0.165
Room allocation not based on IPC principles	2.574	2.499	−0.074	73.7%	0.138
Patient emergency/clinical deterioration	2.418	2.488	0.069	65.3%	0.118
Inadequate IPC education/knowledge	2.490	2.448	−0.042	68.7%	0.386
Lack of hospital management support for resources	2.585	2.436	−0.149	67.8%	0.002
Unexpected increase in patient volume or acuity	2.282	2.418	0.136	61.0%	0.003

**Table 5:** Largest changes in reported reasons for missed infection-control nursing care.

Reason	Group A Mean	Group B Mean	Mean Difference	Important Reason Group A (%)	Important Reason Group B (%)	*p*-Value
Insufficient clerical support	1.468	1.666	+0.198	30.7%	32.4%	0.002
Insufficient physicians	1.879	2.058	+0.179	41.6%	48.3%	0.002
Lack of hospital management support for resources	2.585	2.436	−0.149	75.1%	67.8%	0.002
Unexpected increase in patient volume or acuity	2.282	2.418	+0.136	55.2%	61.0%	0.003
Insufficient storage for personal items	2.136	2.270	+0.134	49.8%	55.4%	0.013
Lack of nursing monitoring of IPC actions	2.497	2.384	−0.113	71.5%	64.0%	0.024
Sterile materials/equipment unavailable when needed	2.381	2.274	−0.107	69.3%	66.0%	0.064
Lack of management support for Infection Control Committees	2.398	2.293	−0.106	65.8%	60.4%	0.053
No chart reminders for infection signs	2.380	2.281	−0.099	63.2%	59.2%	0.062
Ward culture does not support IPC practices	2.362	2.264	−0.098	68.2%	63.4%	0.104

**Table 6:** Multivariable item-level analysis for frequent/always missed infection-control nursing care.

Item	Frequent/Always Omitted Group A (%)	Frequent/Always Omitted Group B (%)	Adjusted OR for Group B	95% CI	*p*-Value
Urinary catheter care every 8 h	43.9%	33.5%	0.72	0.53–0.98	0.037
Disinfection of intravenous access hubs	41.4%	34.6%	0.91	0.69–1.20	0.489
Cleaning bedside table before meal tray	39.8%	30.4%	0.81	0.60–1.09	0.165
Oral hygiene at least once daily	35.2%	31.1%	0.78	0.58–1.05	0.104
Screening new admissions for MDRO infection/colonization	42.7%	28.8%	0.56	0.41–0.76	<0.001
Patient hand hygiene after bedpan/urinal use	26.3%	20.1%	0.92	0.66–1.27	0.601
Hand hygiene before medication administration	22.8%	14.5%	0.70	0.50–0.98	0.036
Gloves during antibiotic preparation/administration	30.2%	16.4%	0.53	0.39–0.73	<0.001
Eye/mask/face shield for airborne or droplet precautions	21.2%	14.1%	0.66	0.47–0.94	0.023
Cleaning/support staff wearing appropriate PPE	18.6%	10.0%	0.58	0.39–0.87	0.008

OR—odds ratio; CI—confidence interval; MDRO—multidrug-resistant organism; PPE—personal protective equipment. Each row represents a separate multivariable logistic regression model. The dependent variable was frequent/always omitted care versus never/rarely/occasionally omitted care for the specific item. Group A was the reference category. Models were adjusted for age, sex, country of work, workplace sector, postgraduate education, specialized IPC duties, previous IPC training, current workplace experience > 10 years, overtime during the previous three months, and perceived impact of insufficient staffing.

## Data Availability

The data are not publicly available due to privacy and ethical restrictions. The dataset contains participant-level responses from nurses working in healthcare settings in Greece and Cyprus, including demographic and professional information. Although the responses were collected anonymously, public sharing of the full dataset was not included in the ethical approvals or participant information process. Therefore, to protect participant confidentiality and comply with the approved ethical framework, the full dataset cannot be made publicly available.
